# Polymorphisms in miR-135a-2, miR-219-2 and miR-211 as well as their interaction with cooking oil fume exposure on the risk of lung cancer in Chinese nonsmoking females: a case–control study

**DOI:** 10.1186/s12885-016-2784-1

**Published:** 2016-09-23

**Authors:** Zhihua Yin, Zhigang Cui, Hang li, Yangwu Ren, Biyun Qian, Nathaniel Rothman, Qing Lan, Baosen Zhou

**Affiliations:** 1 Department of Epidemiology, School of Public Health, China Medical University, No. 77 Puhe Road, Shenyang North New Area, Shenyang, 110122 China; 2 Key Laboratory of Cancer Etiology and Intervention, University of Liaoning Province, No. 77 Puhe Road, Shenyang North New Area, Shenyang, 110122 China; 3 School of Nursing, China Medical University, No. 77 Puhe Road, Shenyang North New Area, Shenyang, 110122 China; 4 Department of Epidemiology, School of Public Health, Shanghai Jiao Tong University, No. 800 Dongchuan Road, Shanghai, 200240 China; 5 Division of Cancer Epidemiology and Genetics, National Cancer Institute, 9000 Rockville Pike, Bethesda, MD 20892 USA

## Abstract

**Background:**

The associations between microRNAs and lung cancer have received increasing attention. This study assess the association between polymorphisms in miR-135a-2, miR-219-2 and miR-211 genes and the risk of lung cancer, as well as the gene–environment interaction between these polymorphisms and cooking oil fume exposure.

**Methods:**

A case–control study featuring 268 cases and 266 controls was conducted. The associations of miR-135a-2 rs10459194, miR-219-2 rs10988341 and miR-211 rs1514035 polymorphisms with the risk of lung cancer were analyzed. The gene–environment interactions were also reported on both additive and multiplicative scales.

**Results:**

There were no statistically significant associations between the single-nucleotide polymorphisms (SNPs) and lung cancer or lung adenocarcinoma. The individuals with both a risk genotype of miRNA SNPs and exposure to a risk factor (cooking oil fumes) were at higher risk of lung cancer than those with only one of these two risk factors (odd ratios of 2.208, 1.285 and 1.813 for miR-135a-2 rs10459194; 2.164, 1.209 and 1.806 for miR-219-2 rs10988341; and 2.122, 1.146 and 1.725 for miR-211 rs1514035, respectively). However, the measures of biological interaction indicate that there was no such interaction between the three SNPs and exposure to cooking oil fumes on an additive scale. Logistic regression models also suggested that the gene–environment interactions were not statistically significant on a multiplicative scale.

**Conclusions:**

There were no significant associations between the polymorphisms in miRNAs (miR-26a-1 rs7372209, miR-605 rs2043556 and miR-16-1 rs1022960) and the risk of lung cancer in the Chinese nonsmoking female population. The interactions between these polymorphisms in miRNAs and cooking oil fume exposure were also not statistically significant.

## Background

Lung cancer is the most commonly diagnosed cancer and the leading cause of cancer-related mortality, causing approximately 1.38 million deaths around the world annually [1]. Although it is acknowledged that smoking is the factor that makes the largest contribution to the risk of lung cancer, approximately 15–25 % of lung cancer patients globally are nonsmokers and the proportion of nonsmokers in female lung cancer patients is as high as 53 % [2, 3], suggesting that other factors such as a genetic predisposition contribute to susceptibility to this disease.

MicroRNA (miRNA) is a class of highly evolutionarily conserved noncoding RNA 18–25 nucleotides in length, which accounts for 1–5 % of the human genome [4] and regulates the expression of approximately >60 % of protein-coding genes. Data indicate that miRNA is involved in almost all important cellular biological processes, including proliferation, stress resistance, apoptosis and differentiation, and that abnormalities in one of these processes may result in a tumor [5, 6]. It has been suggested that a single miRNA can influence the expression of a variety of cancer-related genes. The process by which this occurs involves miRNA binding to the 3′-untranslated region of messenger RNA (mRNA), resulting in the degradation of mRNA or the suppression of its translation into a protein [5, 7]. A difference in the expression level of miRNA between cancerous tissue and adjacent healthy tissue was observed in previous studies, suggesting that miRNA plays a role in tumorigenesis as a tumor suppressor or oncogene, depending on the context.

Single-nucleotide polymorphisms (SNPs) in miRNA gene-coding regions may affect the capacity of miRNA to bind to target mRNA and the maturation of miRNA [8]. Accumulating evidence shows that miRNA SNPs are associated with the risk and prognosis of lung cancer and have great potential to be biomarkers for screening populations at high risk for lung malignancies. A large number of miRNA SNPs associated with lung cancer have been identified. For example, Vinci et al. reported that the CG genotype of miR-146a can increase the risk for non-small cell lung cancer [9]. In addition, Xu et al. determined that the mir-196a2 polymorphism is associated with lung cancer risk [10]. Against this background, based on miRNA and mRNA expression data as well as miRNA–target binding data extracted from the European Bioinformatics Institute(EBI) and the Gene Expression Omnibus (GEO), among others, and using bioinformatic methods such as CN2-SD and a review of the literature, three miRNA SNPs (miR-135a-2 rs10459194, miR-219-2 rs10988341 and miR-211 rs1514035) were selected to investigate their association with lung cancer. To the best of our knowledge, no studies have investigated this association.

Among Chinese female lung cancer patients, who have a low smoking rate [11], smoking is not the main environmental risk factor contributing to lung cancer. Increasing evidence from epidemiological studies on lung cancer shows that exposure to cooking oil fumes is a major environmental factor that may increase the risk of lung cancer in Chinese nonsmoking females [12, 13]. The traditional Chinese cooking style involves stir-frying and deep-frying, which generates oil fumes. Our research team has performed a series of studies about the risk factors for lung cancer in Chinese nonsmoking females since the 1990s, which identified cooking oil fume exposure as a significant risk factor [14–17]. Therefore, in the present study, we explored the interaction of cooking oil fume exposure and miRNA SNPs on the risk of lung cancer. Specifically, here we investigated the relationship of three miRNA SNPs (miR-135a-2 rs10459194, miR-219-2 rs10988341 and miR-211 rs1514035) with the risk of lung cancer and the effect of this combination of miRNA SNPs and cooking oil fumes on this risk in nonsmoking females.

## Methods

This hospital-based case–control study was carried out in Shenyang City, northeast China. It featured 268 female lung cancer patients as cases enrolled from The First Affiliated Hospital of China Medical University and Liaoning Cancer Hospital & Institute. The exclusion criteria for cases were as follows: 1) previous cancer, 2) metastasized cancer, 3) previous radiotherapy or chemotherapy, and 4) smoked more than 100 cigarettes in their lifetime. The control group consisted of 266 cancer-free individuals who were nonsmoking and recruited from medical examination centers during the same period. All subjects were Chinese Han women. The calculated sample sizes were 246, 220 and 224 for miR-135a-2 rs10459194, miR-219-2 rs10988341 and miR-211 rs1514035, respectively. Therefore, the sample size of the present study was sufficient. This study was approved by the Institutional Review Board of China Medical University and written informed consent was obtained from each participant. We interviewed each participant to obtain their demographic data and environmental exposure status when they were admitted to hospital and donated 10 ml of venous blood. With regard to cooking oil fume exposure, participants were asked, “How often does the air in your kitchen become filled with oily smoke during cooking?” There were four possible responses: “never,” “seldom,” “sometimes” and “frequently.” Exposure to cooking oil fumes was categorized as an indicator variable equal to 1 if participants reported frequently or sometimes, and equal to 0 otherwise.

We isolated genomic DNA from the samples of venous blood using the phenol–chloroform method. We performed SNP genotyping by a method that we described previously [18].

The differences in demographic variables and genotype distribution between cases and controls were tested by Student’s *t*-test and *χ*^2^ test. The associations between SNPs and the risk of lung cancer and lung adenocarcinoma were evaluated using the ORs and their 95 % confidence intervals (CIs) calculated by unconditional logistic regression analysis. The relationships of combinations of SNPs and exposure to a range of environmental factors including cooking oil fumes with lung cancer and lung adenocarcinoma were evaluated in the same way. The gene–environment interaction was evaluated using crossover analysis (additive interaction) and logistic regression models (multiplicative interaction). We used those with both the protective genotype and no environmental exposure as a reference group in the analysis. In accordance with a report by Andersson, we calculated three measures of biological interaction: the relative excess risk due to interaction (RERI), the attributable proportion due to interaction (AP) and the synergy index (S), as well as their 95 % CIs based on the three relative risk estimates and the corresponding covariance matrix from a logistic regression model [19]. In the analysis of multiplicative interaction by logistic regression models, only the interaction term (cooking oil fume exposure × the genotype of the studied SNP) was included in the models. SPSS software (Version 20.0; IBM SPSS, Inc., Chicago, IL, USA) was used to perform the statistical analyses mentioned above. All tests were two-sided and statistical significance was defined at *P* < 0.05.

## Results

### Subject characteristics

The present study consisted of 268 cases and 266 controls, who were all nonsmokers. The mean ages of the cases and controls were 55.30 ± 11.85 and 56.71 ± 11.69 years (mean ± SD), respectively. The results of Student’s *t*-test for age indicated no significant difference between these two groups (t = 1.382, *P* = 0.167). The pathological types of lung cancer in the cases were as follows: 197 adenocarcinoma, 44 squamous cell lung cancer and 27 other. The numbers of cases and controls with a history of cooking oil fume exposure were 100 (37.3 %) and 66 (24.8 %); the incidence of exposure was higher in cases than in controls (*χ*^2^ = 9.739, *P* = 0.002). Those exposed to cooking oil fumes had a 1.80-fold higher risk of lung cancer than those without such exposure (OR = 1.80, 95 % CI = 1.24–2.62). The association of other environmental risk factors such as passive smoking and the presence of an indoor ventilation system with lung cancer was not statistically significant (data not shown). The genotype distributions of the three miRNA SNPs (miR-135a-2 rs10459194, miR-219-2 rs10988341 and miR-211 rs1514035) in the cases and controls are shown in Table 1. The genotype frequencies observed in the controls did not diverge significantly from those expected under Hardy–Weinberg equilibrium (*P* = 0.384 for rs10459194, *P* = 0.152 for rs10988341 and *P* = 0.246 for rs1514035).

**Table 1:** Allele and genotype frequencies of miRNA polymorphisms among cases and controls as well as their effects on lung cancer and adenocarcinoma risk in non-smoking females

SNP	No of controls (%)	Lung cancer			Lung adenocarcinoma		
		No (%)	OR [95 % CI]	*P* value	No (%)	OR [95 % CI]	*P* value
miR-135a-2 rs10459194							
TT	219 (82.3)	210 (78.4)	1.00 (ref)		151 (76.6)	1.00 (ref)	
TC	46 (17.3)	56 (20.9)	1.270 (0.823–1.959)	0.281	45 (22.8)	1.419 (0.895–2.248)	0.136
CC	1 (0.4)	2 (0.7)	2.086 (0.188–23.174)	0.550	1 (0.5)	1.450 (0.895–2.248)	0.793
TC + CC vs TT			1.287 (0.838–1.976)	0.249		1.419 (0.899–2.240)	0.132
CC vs TT + TC			1.992 (0.180–22.106)	0.574		1.352 (0.084–21.749)	0.831
T allele	484 (91.0)	476 (88.9)	1.00 (ref)		347 (88.1)	1.00 (ref)	
C allele	48 (9.0)	60 (11.2)	1.271 (0.852–1.896)	0.239	47 (11.9)	1.366 (0.893–2.089)	0.150
miR-219-2 rs10988341							
AA	195 (73.3)	186 (69.4)	1.00 (ref)		136 (69.0)	1.00 (ref)	
AG	62 (23.3)	73 (27.2)	1.234 (0.833–1.830)	0.294	52 (26.4)	1.203 (0.783–1.846)	0.399
GG	9 (3.4)	9 (3.4)	1.048 (0.407–2.699)	0.922	9 (4.6)	1.434 (0.555–3.706)	0.457
AG + GG vs AA			1.211 (0.831–1.763)	0.319		1.232 (0.821–1.849)	0.314
GG vs AA + AG			0.992 (0.388–2.540)	0.987		1.367 (0.532–3.510)	0.516
A allele	452 (85.0)	445 (83.0)	1.00 (ref)		324 (82.2)	1.00 (ref)	
G allele	80 (15.0)	91 (17.0)	1.155 (0.833–1.603)	0.387	70 (17.8)	1.221 (0.859–1.734)	0.265
miR-211 rs1514035							
AA	213 (80.1)	219 (81.7)	1.00 (ref)		165 (83.8)	1.00 (ref)	
AG	48 (18.0)	43 (16.0)	0.871 (0.554–1.370)	0.551	28 (14.2)	0.753 (0.453–1.252)	0.274
GG	5 (1.9)	6 (2.2)	1.167 (0.351–3.882)	0.801	4 (2.0)	1.033 (0.273–3.906)	0.962
AG + GG vs AA			0.899 (0.584–1.385)	0.630		0.779 (0.481–1.264)	0.312
GG vs AA + AG			1.195 (0.360–3.965)	0.770		1.082 (0.287–4.082)	0.908
A allele	474 (89.1)	481 (89.7)	1.00 (ref)		358 (90.9)	1.00 (ref)	
G allele	58 (10.9)	55 (10.3)	0.934 (0.633–1.380)	0.733	36 (9.1)	0.822 (0.530–1.273)	0.379

*Abbreviation*: *SNP* single nucleotide polymorphism, *OR* odds ratio, *CI* confidence interval

### SNP frequencies and associations with lung cancer and lung adenocarcinoma

The relationships of the three miRNA SNPs with susceptibility to lung cancer and lung adenocarcinoma are shown in Table 1. We failed to find any statistically significant associations. It appears that the numbers of carriers with certain SNP genotypes (miR-135a-2 rs10459194 CC, miR-219-2 rs10988341GG and miR-211 rs1514035 GG genotype) were too small to obtain sufficient statistical power.

Table 2 shows the results of crossover analysis of the interaction between exposure to cooking oil fumes and the three miRNA SNPs on lung cancer risk; we found that carriers of the miR-135a-2 rs10459194 TT genotype who had been exposed to cooking oil fumes had a higher risk of lung cancer than such carriers with no such exposure (OR = 1.813, 95 % CI = 1.194–2.753, *P* = 0.005). An identical result was found in the group combining TC and CC carriers (OR = 2.208, 95 % CI = 1.078–4.524, *P* = 0.030). Carriers of the miR-219-2 rs10988341 AA genotype with cooking oil fume exposure were also found to have a higher risk of lung cancer than AA carriers without such exposure (OR = 1.806, 95 % CI = 1.162–2.808, *P* = 0.009). In the group combining those with the AG and GG genotypes, a similar result was obtained (OR = 2.164, 95 % CI = 1.153–4.061, *P* = 0.016). In addition, for miR-211 rs1514035, individuals with the AA genotype and cooking oil fume exposure had a 2.122-fold higher risk of lung cancer than the group combining AG and GG carriers (OR = 2.122, 95 % CI = 1.149–3.921, *P* = 0.016).

**Table 2:** Interaction between SNPs in miRNAs and cooking oil exposure on lung cancer susceptibility in Chinese non-smoking female population

SNP	Oil	No of controls (%)	No of cases (%)	OR (95 % CI)	*P* value
rs10459194					
TT	Non-exposure	166 (62.4)	133 (49.6)	1.00 (ref)	—
TC + CC	Non-exposure	34 (12.8)	35 (13.1)	1.285 (0.761–2.170)	0.349
TT	Exposure	53 (19.9)	77 (28.7)	1.813 (1.194–2.753)	0.005
TC + CC	Exposure	13 (4.9)	23 (8.6)	2.208 (1.078–4.524)	0.030
rs10988341					
AA	Non-exposure	147 (55.3)	117 (43.7)	1.00 (ref)	—
AG + GG	Non-exposure	53 (19.9)	51 (19.0)	1.209 (0.767–1.905)	0.413
AA	Exposure	48 (18.0)	69 (25.7)	1.806 (1.162–2.808)	0.009
AG + GG	Exposure	18 (6.8)	31 (11.6)	2.164 (1.153–4.061)	0.016
rs1514035					
AG + GG	Non-exposure	36 (13.5)	27 (10.1)	1.00 (ref)	—
AA	Non-exposure	164 (61.7)	141 (52.6)	1.146 (0.663–1.982)	0.625
AG + GG	Exposure	17 (6.4)	22 (8.2)	1.725 (0.771–3.963)	0.185
AA	Exposure	49 (18.4)	78 (29.1)	2.122 (1.149–3.921)	0.016

In the subgroup of those with lung adenocarcinoma, the results were analogous to those in the lung cancer group, as shown in Table 3. Carriers of the miR-135a-2 rs10459194 TT genotype, the miR-219-2 rs10988341 AA genotype and the miR-211 rs1514035 AA genotype with exposure to cooking oil fumes had a higher risk of lung adenocarcinoma (rs10459194 TT: OR = 1.597, 95 % CI = 1.011–2.524; rs10988341 AA: OR = 1.806, 95 % CI = 1.162–2.808; rs1514035 AA: OR = 2.167, 95 % CI = 1.115–4.215) The significant results were also found in the group combining miR-135a-2 rs10459194 TC and CC carriers, and the group combining miR-219-2 rs10988341 AG and GG carriers with cooking oil fume exposure (rs10459194 TC + CC: OR = 2.554, 95 % CI = 1.217–5.358; rs10988341 AG + GG: OR = 2.065, 95 % CI = 1.049–4.064).

**Table 3:** Interaction between SNPs in miRNAs and cooking oil exposure on lung adenocarcinoma in Chinese non-smoking female population

SNP	Oil	No of controls (%)	No of cases (%)	OR (95 % CI)	*P* value
rs10459194					
TT	Non-exposure	166 (62.4)	100 (50.8)	1.00 (ref)	—
TC + CC	Non-exposure	34 (12.8)	26 (13.2)	1.269 (0.720–2.240)	0.410
TT	Exposure	53 (19.9)	51 (25.9)	1.597 (1.011–2.524)	0.045
TC + CC	Exposure	13 (4.9)	20 (10.2)	2.554 (1.217–5.358)	0.013
rs10988341					
AA	Non-exposure	147 (55.3)	87 (44.2)	1.00 (ref)	—
AG + GG	Non-exposure	53 (19.9)	39 (19.8)	1.243 (0.761–2.032)	0.385
AA	Exposure	48 (18.0)	49 (24.9)	1.725 (1.069–2.783)	0.025
AG + GG	Exposure	18 (6.8)	22 (11.2)	2.065 (1.049–4.064)	0.036
rs1514035					
AG + GG	Non-exposure	36 (13.5)	20 (10.2)	1.00 (ref)	—
AA	Non-exposure	164 (61.7)	106 (53.8)	1.163 (0.639–2.117)	0.620
AG + GG	Exposure	17 (6.4)	12 (6.1)	1.271 (0.507–3.186)	0.610
AA	Exposure	49 (18.4)	59 (29.9)	2.167 (1.115–4.215)	0.023

**Table 4:** Interaction measures between SNPs in miRNAs and cooking oil exposure on lung cancer and adenocarcinoma in Chinese non-smoking female population

SNP	Lung cancer			Lung adenocarcinoma		
	Measure	Estimate	95 % CI	Measure	Estimate	95 % CI
rs10459194						
	RERI	0.110	−1.641, 1.861	RERI	0.687	−1.309, 2.683
	AP	0.050	−0.716, 0.815	AP	0.269	−0.357, 0.895
	S	1.100	0.247, 4.906	S	1.793	0.363, 8.848
rs10988341						
	RERI	0.149	−1.362, 1.659	RERI	0.097	−1.468, 1.662
	AP	0.069	−0.599, 0.736	AP	0.047	−0.689, 0.783
	S	1.147	0.291, 4.154	S	1.100	0.239, 5.067
rs1514035						
	RERI	0.251	−1.154, 1.656	RERI	0.733	−0.554, 2.021
	AP	0.118	−0.541, 0.777	AP	0.338	−0.237, 0.914
	S	1.287	0.256, 6.463	S	2.690	0.117, 61.648

*RERI* relative excess risk due to interaction, *AP* attributable proportion due to interaction, *S* synergy index, *95 % CI* 95 % confidence interval

Above cross-over results indicated that the gene-environment interaction may exist, so statistical tests were used to evaluate the significance of the interaction on both additive scale and multiplicative scale. Table 4 showed the interaction results on an additive scale including three measures and their 95 % CIs to suggest the biological interaction. The results suggested that the interactions between the SNPs and cooking fume exposure were not significant on an additive scale. In the analysis of gene–environment interaction, logistic models suggested that the gene–environment interaction was not statistically significant on a multiplicative scale. In logistic regression analyses of lung cancer, ORs (95 % CIs) and *P*-values of interaction terms were 0.948 (0.375–2.395) and 0.910 for oil × rs10459194, 0.991 (0.434–2.260) and 0.983 for oil × rs10988341, and 1.073 (0.432–2.665) and 0.879 for oil × rs1514035, respectively. In adenocarcinoma, ORs (95 % CIs) and *P*-values of interaction terms were 1.259 (0.473–3.351) and 0.644 for oil × rs10459194, 0.963 (0.396–2.339) and 0.934 for oil × rs10988341, and 1.466 (0.527–4.081) and 0.464 for oil × rs1514035, respectively.

## Discussion

The etiopathogenesis of lung cancer is a complicated issue in which multiple factors are involved. Our understanding of the pathogenic mechanisms in the carcinogenesis of lung malignancies is still limited. Smoking has been established as a predominant environmental risk factor for lung cancer, but the prevalence of smoking is very low in Chinese women (2.4 % for those over 15 years old), according to a report published by the World Health Organization [11]; in addition, it was reported that 53 % of female lung cancer patients were nonsmokers [2]. Therefore, for Chinese females, there may be environmental risk factors other than smoking that make larger contributions to lung cancer susceptibility. The traditional Chinese style of cooking often involves stir-frying and deep-frying, in which oil is usually heated to a high temperature and some mutagens and human carcinogens such as polycyclic aromatic hydrocarbons and benzo[a]pyrene 7,8-diol 9,10-epoxide are generated, which may result in DNA damage to the cells, thus increasing susceptibility to lung cancer [20]. Our research team has performed studies to verify the significant associations between cooking oil fume exposure and lung cancer risk in Chinese nonsmoking females [14–17, 21, 22]. The present study suggested that individuals with exposure to cooking oil fumes had a 1.80-fold increased risk of developing lung cancer. It was also reported that cooking oil fume condensates could lead to DNA damage [23], induce an increase of DNA crosslinks [24], and inhibit cell growth and increase TGF-β1 secretion, resulting in oxidative stress in lung epithelial cells [25]. Other population-based studies have also shown the importance of cooking oil fume exposure in the risk of lung cancer among nonsmoking females [26–28].

The relationship between mutations such as SNPs in miRNA gene-coding regions and cancer susceptibility has become a major focus of attention in cancer research in recent decades. miRNAs play a role as tumor suppressors or oncogenes in malignancies, and accumulating evidence from miRNA expression profiles has demonstrated the ectopic expression of miRNAs in malignant tissues compared with that in adjacent nontumor tissue [29–31]. In addition, SNPs in miRNA-coding regions may affect the expression level of miRNAs, thus potentially having an effect on susceptibility to lung cancer. This background prompted us to evaluate the associations between three miRNA SNPs and lung cancer risk, but we did not obtain any statistically significant results. In nonsmokers, as lung adenocarcinoma is the most common type of lung cancer, so we subsequently conducted a subgroup analysis stratified by histopathological type, but also obtained no statistically significant results. We attribute this to the small numbers of carriers of some genotypes, so a larger sample size may provide sufficient statistical power to validate such association.

For miR-135a-2 rs10459194, TT carriers and the group combining CT and CC carriers who had been exposed to cooking oil fumes were found to have a greater risk of lung cancer than carriers of the TT genotype without such exposure. This is consistent with evidence that cooking oil fume exposure may increase the risk of developing lung cancer. In addition, specifically in lung adenocarcinoma, an elevated risk was also observed in TT carriers and the group combining CT and CC carriers with cooking oil fume exposure. Abnormal expression of miR-135a has been observed in several kinds of malignancy, suggesting its role in carcinogenesis. For example, a study by Navarro et al. found that miR-135a can function as a tumor suppressor by targeting JAK2 to suppress STAT3 activation, and showed that the expression of cyclin D1 and Bcl-xL was reduced in classic Hodgkin’s lymphoma. This was supported by similar findings in another study by Wu et al. that investigated the role of miR-135a in gastric cancer [32]. However, to the best of our knowledge, no previous studies focused on the association between miR-135a-2 rs10459194 and lung cancer risk. We thus believe that this is the first study to evaluate the association between this novel miRNA SNP and susceptibility to lung malignancies.

The biological functions of miR-211 in the carcinogenesis of a variety of malignancies have been extensively investigated, with the results suggesting that it can act as a tumor suppressor or oncogene depending on the tissue and other characteristics. In an in vitro study by Boyle et al., it was reported that the overexpression of miR-211 may decrease cancer invasiveness by directly targeting BRN2 translation in melanoma cells [33]. However, in another in vitro study that investigated the role of miR-211 in colorectal cancer, it was found that the enforced expression of miR-211 can promote cancer cell growth by suppressing the expression of CHD5, which is a tumor suppressor [34]. Here, we report for the first time that miR-211 rs1514035 AA genotype carriers who had been exposed to cooking oil fumes had an increased risk of developing lung cancer compared with the group combining AG and GG genotype carriers without such exposure. As an example of another miRNA that might play important roles in cancer, miR-219 has been reported to be involved in the carcinogenesis of a range of malignancies. A study by Lei et al. indicated that its expression was downregulated in gastric cancer specimens, and that reintroducing the expression of miR-219 may suppress cell proliferation, migration and invasion and induce apoptosis, suggesting that miR-219 plays a tumor suppressor role in gastric cancer [35]. In addition, in hepatocellular carcinoma, miR-219 also acts as a tumor suppressor that was found to be significantly downregulated and to be able to suppress cell proliferation by targeting glypican-3 [36]. However, no studies have reported the biological function of miR-219 in the carcinogenesis of lung cancer. In the current study, we observed a statistically significant increase in the risk of lung cancer and lung adenocarcinoma in those with the miR-219-2 rs10988341 AA genotype and the group combining AG and GG genotype carriers who had also been exposed to cooking oil fumes.

The effects of gene–environment interaction on lung cancer risk have seldom been investigated. Hence, in the present study, we evaluated the interaction between exposure to cooking oil fumes and three miRNA SNPs on lung cancer risk. However, we did not obtain any statistically significant results for this interaction, which may be attributable to the small sample size that precluded sufficient statistical power being obtained. Therefore, the effects of this gene–environment interaction on susceptibility to lung cancer should be investigated in further studies with larger samples.

There are several limitations of the current study that should be noted. First, this is a hospital-based case–control study in which the subjects were enrolled from hospitals, which may have resulted in selection bias and prevented the sample from being approximately representative of the overall population. As such, caution should be taken when extrapolating the findings to other populations prior to validation in larger samples. Second, all the results in the present study were only statistically significant, the underling mechanisms remain unknown. Third, the sample size may have been too small to obtain conclusive results, so the findings need to be validated in further studies.

## Conclusions

In this study, no significant associations were identified between miRNA polymorphisms (miR-26a-1 rs7372209, miR-605 rs2043556, miR-16-1 rs1022960) and the risk of lung cancer in a Chinese nonsmoking female population. In addition, the interactions between these miRNA polymorphisms and cooking oil fume exposure were not statistically significant.

## Abbreviations

AP: The attributable proportion due to interaction
CI: Confidence interval
mRNA: Message RNA
OR: Odds ratio
RERI: The relative excess risk due to interaction
S: The synergy index
SNP: Single nucleotide polymorphism
